# Editorial: The Sociology of Vaccines

**DOI:** 10.12688/f1000research.124587.1

**Published:** 2022-08-04

**Authors:** Michael Calnan, Jens O. Zinn, Tom Douglass

**Affiliations:** 1 School of Social Policy, Sociology and Social Research, University of Kent, Cornwallis East, Canterbury, Kent, CT2 7NF, UK; 2 School of Social and Political Sciences, University of Melbourne, Parkville, Victoria, 3010, Australia; 3 School of Social Work and Social Care, Park House, University of Birmingham, Edgbaston, Birmingham, B15 T22, UK

**Keywords:** Vaccines, Sociology, Risk, Uncertainty, Pharmaceutical Industry, Vaccine Development

## Abstract

In this editorial, we identify the key questions requiring further exploration in the sociology of vaccines. In doing so, we discuss the socio-structural forces shaping views towards knowledge about and access to vaccination, trust in vaccines and regulators/decision makers, the associated problem of financial interests in vaccine development and regulation, and global vaccine inequalities. Across the breadth of these issues, we additionally identify a range of theoretical perspectives and conceptual directions that sociologists might utilise when producing innovative empirical, methodological and theoretical research on vaccination relating to risk and uncertainty, conflicts of interest, power and inequality.

Vaccines have been portrayed as one of the success stories of Western scientific medicine. In this narrative, vaccines have helped to manage and conquer deadly and debilitating diseases ( Calnan & Douglass, 2020). Despite their apparent overall success, vaccines are associated with a long history of controversy, hesitancy, and resistance in which they connect to broader social issues, such as modes of governance, questions of justice in the face of systematic social inequalities, the social influence of science as well as the trustworthiness of decision makers and the pharmaceutical industry. It is our aim in this collection to encourage sociologists and social scientists more broadly to advance understanding of the social processes shaping the production, governance, and acceptance of vaccines in a globalising world through empirical and theoretical research.

For many years, people in countries globally have resisted compulsory vaccination as a violation of personal liberty ( Calnan & Douglass, 2020). Instead, the liberal democracies of the Global North developed voluntary vaccination programmes which successfully created the high vaccination rates necessary to reach herd immunity and conquer disease ( Haverkate *et al.*, 2012; Vanderslott & Marks, 2021). However, in more recent times, the wealthy Global North has witnessed slowly declining willingness to vaccinate ( Trust, 2019). When, as a result, illnesses such as measles returned governments responded strongly with compulsory measures ( Brady, 2019). A mix of explanations have been suggested for the long-term decrease in people’s willingness to accept vaccination – or, in other words, people’s increasing vaccine hesitancy. This includes the growing dominance of neoliberal policies supporting individualised rather than collective health solutions with alternative approaches, such as homeopathy, becoming more influential ( Hobson-West, 2003). Health controversies are additionally associated with this trend, such as the MMR scandal in the UK, raising doubts in regulators and vaccine safety ( Hobson-West, 2007) as well as a growing role for social media in spreading misinformation and conspiracy theories ( Cinelli *et al.*, 2020). Theorising of a shift in governmentality to neoliberalism as well as the cultural approach to risk analysis highlighting competing worldviews feeding risk conflicts are valuable scholarly resources to make sense of these complex short-term and long-term developments and different responses to the provision of vaccines.

There is good evidence that the take up vaccination is shaped by socio-structural forces. These forces not only produce different worldviews but present disparate vaccination opportunities and availability, foster distinctive information and knowledge about vaccination, and create a range of priorities when managing a life at risk. The cultural approach to the analysis of risk suggests that people at the margins of society often doubt science, and deeply distrust government and state institutions. Meanwhile, some feminist scholars have shown that disadvantage is best understood as the intersections of various forces such as gender, social class, and race, amongst others ( Giritli Nygren *et al.*, 2020; Olofsson *et al.*, 2014). The nature of how these intersecting forces influence knowledge about and access to vaccines requires further exploration.

Risk always comes with uncertainties which challenge decision makers as well as people in everyday life to find the right balance to overreact or underestimate uncertain dangers ( Giddens, 2000). The risks of a disease must be balanced against the risks of a vaccine. At times of crisis, the history of vaccines (and the pharmaceutical sector more generally) has occasionally witnessed the premature release of products with unexpected side-effects causing more harm than the disease ( Silverstein, 1981). At the same time overestimation of possible harm of disease, leading to large overspending on ineffective vaccines, has been criticised for the possible conflicts of interest of decision makers promoting vaccination ( Holland *et al.*, 2014). Parallel to the increasing medicalisation of societies arrive concerns about the supposedly altruistic motives of vaccine producers and concerns about the failure of regulation in the pharmaceutical sector generally (e.g., as fostered by major disasters such as the Thalidomide scandal in the mid-20 ^th^ century). There has been an erosion of trust in both the pharmaceutical industry and state regulation. Underpinning this erosion of trust, in the UK and USA particularly, is evidence of significant corporate bias and leaning to the interests of the drug industry ( Abraham, 1995, 2009). Sociologists drawing on or influenced by political economy and utilising concepts such as corporate bias are uniquely well placed to analyse how economic interests might shape vaccination regulation and policy.

Since the beginning of the COVID-19 crisis vaccines have been presented as providing the ultimate protection against COVID-19. However, with the virus mutating and the protection lasting only for a limited time it has become increasingly clear that vaccination in this context means mainly lowering the likelihood of severe illness rather than full protection. This raises questions not only about the decision to vaccinate but for the development of costly vaccines, such as who provides the manufacturing resources, who shoulders the burden of the financial risks, as well as who gains from vaccine development. In this regard, there are other salient theoretical approaches that enable scholars to analyse power dynamics and the influence of powerful interests in vaccination policy, such as the theory of countervailing powers (see Calnan and Douglass, 2022). The reconstruction of how complex assemblages of viruses, vaccines, and humans produce the social world of vaccination, their development, distribution and efficient application can also be explored using a material-semiotic approach ( Latour, 2005).

The COVID-19 crisis renewed focus on infectious diseases as an increasing global problem. As global risks, pandemics require global responses and a cosmopolitan worldview ( Beck, 1992, 2009). The World Health Organization (WHO) in its role to identify and inform health threats globally and to provide support moved centre stage during the COVID-19 crisis. It emphasised governments’ cosmopolitan responsibility accusing countries of vaccine nationalism (where countries prioritise their domestic population and stockpile vaccines) branding global inequities in vaccine production and distribution as moral failure ( Lupton, 2022). The fast development of a COVID-19 vaccine contrasts sharply with the slow production of vaccines against Ebola which lasted decades ( Roemer-Mahler & Elbe, 2016) partially reflecting the fact that Ebola never significantly impacted countries of the Global North. Global vaccine inequalities also reflect the rationale, framing and approach to knowledge sharing adopted by the Global North that treating a disease where it occurs in the Global South is the best protection for countries of the Global North. Scholarship utilising post-colonial theory could explore the globally established exchange mechanisms and relationships which shape ongoing understanding and management of vaccines. This would also include analysis of system competition and distrust making cosmopolitan collaboration in vaccine production and dissemination in a competitive world difficult.

In conclusion, this editorial has identified some of the key sociological questions which need to be explored to gain greater insights into how vaccines are produced, allocated, utilised and taken up both nationally and globally. We have additionally discussed a number of different theoretical perspectives and dimensions to highlight some of the possible conceptual directions when sociologically analysing vaccination, with the aim of inspiring new and innovative empirical, methodological and theoretical research in the sociology of vaccines relating particularly to risk and uncertainty, financial interests, power and inequality. In this regard, we hope to influence sociological analysts to engage with a range of new, or underexplored questions within the sociology of vaccines whilst also enabling scholars to find new ways to analyse existing or more well researched problems within the sociology of vaccines.

## Data availability

No data are associated with this article.
